# *catena*-Poly[[[diaqua­bis[4-(diethyl­amino)benzoato-κ*O*]manganese(II)]-μ-aqua] dihydrate]

**DOI:** 10.1107/S1600536809021060

**Published:** 2009-06-06

**Authors:** Tuncer Hökelek, Hakan Dal, Barış Tercan, Özgür Aybirdi, Hacali Necefoğlu

**Affiliations:** a Department of Physics, Hacettepe University, 06800 Beytepe, Ankara, Turkey; b Department of Chemistry, Faculty of Science, Anadolu University, 26470 Yenibağlar, Eskişehir, Turkey; c Department of Physics, Karabük University, 78050 Karabük, Turkey; d Department of Chemistry, Kafkas University, 63100 Kars, Turkey

## Abstract

In the crystal structure of the title complex, {[Mn(C_11_H_14_NO_2_)_2_(H_2_O)_3_]·2H_2_O}_*n*_, the two independent Mn^II^ atoms are located on a centre of symmetry and coordinated by two 4-(diethyl­amino)benzoate (DEAB) anions and two water mol­ecules in the basal plane while another water mol­ecule bridges the Mn atoms in the apical directions, forming polymeric chains. The dihedral angles between the carboxyl­ate groups and the adjacent benzene rings are 11.33 (13) and 10.90 (9)° and the benzene rings are oriented at a dihedral angle of 67.88 (6)°. The uncoordinated water mol­ecules link the carboxyl­ate groups and coordinated water mol­ecules *via* O—H⋯O hydrogen bonding. Weak C—H⋯π inter­actions are also found in the crystal structure.

## Related literature

For the applications of transition metal complexes with biochemical mol­ecules in biological systems, see: Antolini *et al.* (1982 ▶). Benzoic acid derivatives such as 4-amino­benzoic acid are used extensively as bifunctional organic ligands in coordination chemistry due to their various coordination modes, see: Chen & Chen (2002 ▶); Amiraslanov *et al.* (1979 ▶); Hauptmann *et al.* (2000 ▶). In pellagra disease, niacin deficiency leads to loss of copper from the body with high serum and urinary copper levels (Krishnamachari, 1974 ▶). The nicotinic acid derivative *N*,*N*-Diethyl­nicotinamide (DENA) is an important respiratory stimulant (Bigoli *et al.*, 1972 ▶). For structure–function–coordination relationships of the aryl­carboxyl­ate ion in Mn^II^ complexes of benzoic acid derivatives, see: Shnulin *et al.* (1981 ▶); Antsyshkina *et al.* (1980 ▶); Adiwidjaja *et al.* (1978 ▶); Catterick *et al.* (1974 ▶); Bigoli *et al.* (1972 ▶). For related structures, see: Hökelek *et al.* (1995 ▶, 2007 ▶, 2008 ▶); Hökelek & Necefoğlu (1996 ▶, 1997 ▶, 1998 ▶, 2007 ▶).
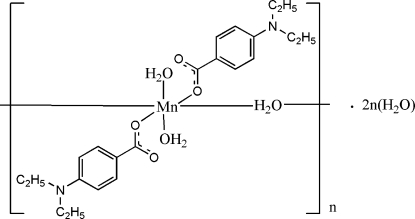


## Experimental

### 

#### Crystal data

[Mn(C_11_H_14_NO_2_)_2_(H_2_O)_3_]·2H_2_O
*M* *_r_* = 529.48
Monoclinic, 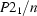

*a* = 8.1585 (2) Å
*b* = 11.2907 (2) Å
*c* = 27.8738 (3) Å
β = 95.644 (2)°
*V* = 2555.15 (8) Å^3^
*Z* = 4
Mo *K*α radiation
μ = 0.57 mm^−1^
*T* = 100 K
0.50 × 0.20 × 0.15 mm

#### Data collection

Bruker Kappa APEXII CCD area-detector diffractometer
Absorption correction: multi-scan (*SADABS*; Bruker, 2005 ▶) *T*_min_ = 0.870, *T*_max_ = 0.920
22676 measured reflections
6299 independent reflections
4556 reflections with *I* > 2σ(*I*)
*R*_int_ = 0.035

#### Refinement

*R*[*F* ^2^ > 2σ(*F* ^2^)] = 0.038
*wR*(*F* ^2^) = 0.091
*S* = 1.02
6299 reflections
354 parameters
15 restraints
H atoms treated by a mixture of independent and constrained refinement
Δρ_max_ = 0.66 e Å^−3^
Δρ_min_ = −0.41 e Å^−3^

### 

Data collection: *APEX2* (Bruker, 2007 ▶); cell refinement: *SAINT* (Bruker, 2007 ▶); data reduction: *SAINT*; program(s) used to solve structure: *SHELXS97* (Sheldrick, 2008 ▶); program(s) used to refine structure: *SHELXL97* (Sheldrick, 2008 ▶); molecular graphics: *ORTEP-3 for Windows* (Farrugia, 1997 ▶); software used to prepare material for publication: *WinGX* (Farrugia, 1999 ▶) and *PLATON* (Spek, 2009 ▶).

## Supplementary Material

Crystal structure: contains datablocks I, global. DOI: 10.1107/S1600536809021060/xu2525sup1.cif

Structure factors: contains datablocks I. DOI: 10.1107/S1600536809021060/xu2525Isup2.hkl

Additional supplementary materials:  crystallographic information; 3D view; checkCIF report

## Figures and Tables

**Table 1:** Selected geometric parameters (Å, °)

Mn1—O2	2.1071 (14)
Mn1—O5	2.1932 (14)
Mn1—O6	2.2725 (13)
Mn2—O4	2.1120 (13)
Mn2—O6	2.2594 (13)
Mn2—O7	2.1835 (14)

**Table 2:** Hydrogen-bond geometry (Å, °)

*D*—H⋯*A*	*D*—H	H⋯*A*	*D*⋯*A*	*D*—H⋯*A*
O5—H51⋯O8	0.97 (2)	1.77 (2)	2.738 (2)	177 (2)
O5—H52⋯O4^iii^	0.902 (17)	1.846 (17)	2.7457 (19)	175 (3)
O6—H61⋯O3	0.929 (17)	1.778 (19)	2.651 (2)	156 (2)
O6—H62⋯O1^i^	0.896 (18)	1.76 (2)	2.608 (2)	156 (3)
O7—H71⋯O9^i^	0.97 (2)	1.77 (2)	2.739 (2)	177.0 (2)
O7—H72⋯O2^ii^	0.893 (18)	1.850 (18)	2.733 (2)	170 (3)
O8—H82⋯O3	0.96 (3)	1.77 (3)	2.694 (2)	160 (3)
O9—H91⋯O1	0.927 (19)	1.80 (2)	2.692 (2)	160 (3)
C6—H6⋯*Cg*2	0.93	2.91	3.764 (2)	154
C19—H19*A*⋯*Cg*2^iv^	0.97	2.90	3.830 (2)	162

Symmetry codes: (i) 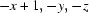; (ii) 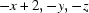; (iii) 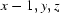; (iv) 
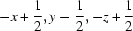
. *Cg*2 is the centroid of the C13–C18 ring.

**Fig. 1.**
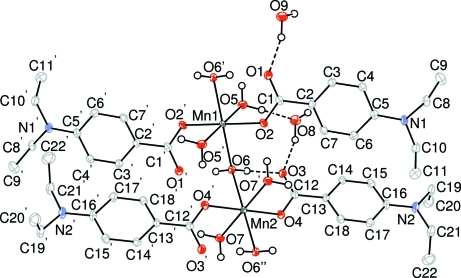
The molecular structure of the title molecule with the atom-numbering scheme. Displacement ellipsoids are drawn at the 50% probability level. Hydrogen bonds are shown as dashed lines. Hydrogen atoms not involved in hydrogen bonding are omitted for clarity. Primed atoms are generated by the symmetry operators: (') 1 - x, -y, -z, ('') 2 - x, -y, -z.

## supplementary crystallographic information

### Comment

Transition metal complexes with biochemical molecules show interesting physical and/or chemical properties, through which they may find applications in biological systems (Antolini *et al.*, 1982). Some benzoic acid derivatives, such as 4-aminobenzoic acid, have been extensively reported in coordination chemistry, as bifunctional organic ligands, due to the varieties of their coordination modes (Chen & Chen, 2002; Amiraslanov *et al.*, 1979; Hauptmann *et al.*, 2000). Nicotinamide (NA) is one form of niacin. A deficiency of this vitamin leads to loss of copper from the body, known as pellagra disease. Victims of pellagra show unusually high serum and urinary copper levels (Krishnamachari, 1974). The nicotinic acid derivative *N*,*N*-Diethylnicotinamide (DENA) is an important respiratory stimulant (Bigoli *et al.*, 1972).

The structure-function-coordination relationships of the arylcarboxylate ion in Mn^II^ complexes of benzoic acid derivatives may also change depending on the nature and position of the substituted groups on the benzene ring, the nature of the additional ligand molecule or solvent, and the pH and temperature of synthesis (Shnulin *et al.*, 1981; Antsyshkina *et al.*, 1980; Adiwidjaja *et al.*, 1978). When pyridine and its derivatives are used instead of water molecules, the structure is completely different (Catterick *et al.*, 1974).

The structure determination of the title compound, (I), a polymeric manganese complex with four 4-diethylaminobenzoate (DEAB) ligands and five coordinated and two uncoordinated water molecules, was undertaken in order to determine the properties of the ligands and also to compare the results obtained with those reported previously.

In the polymeric title complex, (I), each Mn atom is located on a centre of symmetry, and surrounded by two DEAB and four water molecules. The DEAB ligands are monodentate and a water molecule bridges the two Mn atoms (Fig. 1). The four O atoms (O2, O2', O5, O5' and O4, O4', O7, O7' atoms) in the equatorial planes around each Mn atom form a slightly distorted square-planar arrangement, while the slightly distorted octahedral coordination is completed by the symmetry related O atoms of the bridging water molecule (O6, O6' and O6'') in the axial positions (Table 1 and Fig. 1).

The near equality of the C1—O1 [1.263 (2) Å], C1—O2 [1.279 (2) Å], C12—O3 [1.263 (2) Å] and C12—O4 [1.278 (2) Å], bonds in the carboxylate group indicates a delocalized bonding arrangement, rather than localized single and double bonds, and may be compared with the corresponding distances: 1.256 (6) and 1.245 (6) Å in [Mn(DENA)_2_(C_7_H_4_ClO_2_)_2_(H_2_O)_2_], (II) (Hökelek *et al.*, 2008), 1.265 (6) and 1.275 (6) Å in [Mn(C_9_H_10_NO_2_)_2_(H_2_O)_4_]. 2(H_2_O), (III) (Hökelek & Necefoğlu, 2007), 1.260 (4) and 1.252 (4) Å in [Zn(DENA)_2_(C_7_H_4_FO_2_)_2_(H_2_O)_2_],(IV) (Hökelek *et al.*, 2007), 1.259 (9) and 1.273 (9) Å in Cu_2_(DENA)_2_(C_6_H_5_COO)_4_, (V) (Hökelek *et al.*, 1995), 1.279 (4) and 1.246 (4) Å in [Zn_2_(DENA)_2_(C_7_H_5_O_3_)_4_]. 2H_2_O, (VI) (Hökelek & Necefoğlu, 1996), 1.251 (6) and 1.254 (7) Å in [Co(DENA)_2_(C_7_H_5_O_3_)_2_(H_2_O)_2_], (VII) (Hökelek & Necefoğlu, 1997) and 1.254 (2) and 1.251 (2) Å in [Co(NA)_2_(C_7_H_4_NO_4_)_2_(H_2_O)_2_], (VIII) (Hökelek & Necefoğlu, 1998).

In (I), the average Mn—O bond length is 2.1880 (14) Å and the Mn atoms are displaced out of the least-squares planes of the carboxylate groups (O1/C1/O2) and (O3/C12/O4) by -0.857 (1) Å and 1.004 (1) Å, respectively. The dihedral angles between the planar carboxylate groups and the adjacent benzene rings A (C2—C7) and B (C13—C18) are 11.33 (13)° and 10.90 (9)°, respectively, while those between rings A and B are A/B = 67.88 (6)°. Intramolecular O—H···O hydrogen bonds (Table 2) link the uncoordinated water molecules to the carboxylate groups and coordinated water molecules (Fig. 1).

In the crystal structure, strong intra- and intermolecular O—H···O hydrogen bonds (Table 2) link the molecules into a supramolecular structure, in which they may be effective in the stabilization of the structure. There also exist two weak C—H···π interactions (Table 2).

### Experimental

The title compound was prepared by the reaction of MnSO_4_.H_2_O (0.85 g, 5 mmol) in H_2_O (50 ml) and NA (1.22 g, 10 mmol) in H_2_O (50 ml) with sodium *p*-dimethylaminobenzoate (2.16 g, 10 mmol) in H_2_O (100 ml). The mixture was filtered and set aside to crystallize at ambient temperature for one week, giving single crystals.

### Refinement

H atoms of water molecules were located in difference Fourier maps and refined isotropically, with restrains of O5—H51 = 0.970 (16), O5—H52 = 0.903 (17), O6—H61 = 0.931 (16), O6—H62 = 0.896 (18), O7—H71 = 0.973 (16), O7—H72 = 0.894 (17), O8—H81 = 0.895 (17), O8—H82 = 0.957 (19), O9—H91 = 0.930 (17), O9—H92 = 0.90 (2) Å and H51—O5—H52 = 105 (2), H61—O6—H62 = 106 (2), H71—O7—H72 = 106 (2), H81—O8—H82 = 105 (3) and H91—O9—H92 = 105 (3)°. The remaining H atoms were positioned geometrically with C—H = 0.93, 0.97 and 0.96 Å, for aromatic, methylene and methyl H atoms, respectively, and constrained to ride on their parent atoms, with *U*_iso_(H) = *xU*_eq_(C), where *x* = 1.5 for methyl H and *x* = 1.2 for all other H atoms.

### Crystal data

**Table:**

[Mn(C_11_H_14_NO_2_)_2_(H_2_O)_3_]·2H_2_O	*F*(000) = 1124
*M**_r_* = 529.48	*D*_x_ = 1.376 Mg m^−^^3^
Monoclinic, *P*2_1_/*n*	Mo *K*α radiation, λ = 0.71073 Å
Hall symbol: -P 2yn	Cell parameters from 6515 reflections
*a* = 8.1585 (2) Å	θ = 2.3–28.0°
*b* = 11.2907 (2) Å	µ = 0.57 mm^−^^1^
*c* = 27.8738 (3) Å	*T* = 100 K
β = 95.644 (2)°	Block, yellow
*V* = 2555.15 (8) Å^3^	0.50 × 0.20 × 0.15 mm
*Z* = 4	

### Data collection

**Table:**

Bruker Kappa APEXII CCD area-detector diffractometer	6299 independent reflections
Radiation source: fine-focus sealed tube	4556 reflections with *I* > 2σ(*I*)
graphite	*R*_int_ = 0.035
φ and ω scans	θ_max_ = 28.4°, θ_min_ = 1.5°
Absorption correction: multi-scan (*SADABS*; Bruker, 2005)	*h* = −9→10
*T*_min_ = 0.870, *T*_max_ = 0.920	*k* = −11→15
22676 measured reflections	*l* = −37→35

### Refinement

**Table:**

Refinement on *F*^2^	Primary atom site location: structure-invariant direct methods
Least-squares matrix: full	Secondary atom site location: difference Fourier map
*R*[*F*^2^ > 2σ(*F*^2^)] = 0.038	Hydrogen site location: inferred from neighbouring sites
*wR*(*F*^2^) = 0.091	H atoms treated by a mixture of independent and constrained refinement
*S* = 1.01	*w* = 1/[σ^2^(*F*_o_^2^) + (0.0367*P*)^2^ + 1.1841*P*] where *P* = (*F*_o_^2^ + 2*F*_c_^2^)/3
6299 reflections	(Δ/σ)_max_ < 0.001
354 parameters	Δρ_max_ = 0.66 e Å^−^^3^
15 restraints	Δρ_min_ = −0.41 e Å^−^^3^

### Special details

**Table:**

Geometry. All e.s.d.'s (except the e.s.d. in the dihedral angle between two l.s. planes) are estimated using the full covariance matrix. The cell e.s.d.'s are taken into account individually in the estimation of e.s.d.'s in distances, angles and torsion angles; correlations between e.s.d.'s in cell parameters are only used when they are defined by crystal symmetry. An approximate (isotropic) treatment of cell e.s.d.'s is used for estimating e.s.d.'s involving l.s. planes.
Refinement. Refinement of *F*^2^ against ALL reflections. The weighted *R*-factor *wR* and goodness of fit *S* are based on *F*^2^, conventional *R*-factors *R* are based on *F*, with *F* set to zero for negative *F*^2^. The threshold expression of *F*^2^ > σ(*F*^2^) is used only for calculating *R*-factors(gt) *etc*. and is not relevant to the choice of reflections for refinement. *R*-factors based on *F*^2^ are statistically about twice as large as those based on *F*, and *R*- factors based on ALL data will be even larger.

### Fractional atomic coordinates and isotropic or equivalent isotropic displacement parameters (Å^2^)

**Table:**

	*x*	*y*	*z*	*U*_iso_*/*U*_eq_	
Mn1	0.5000	0.0000	0.0000	0.01208 (10)	
Mn2	1.0000	0.0000	0.0000	0.01215 (10)	
O1	0.32484 (17)	−0.26988 (12)	0.00330 (5)	0.0168 (3)	
O2	0.56738 (16)	−0.17869 (12)	0.01144 (5)	0.0184 (3)	
O3	0.86399 (16)	−0.04378 (12)	0.10998 (5)	0.0162 (3)	
O4	1.08825 (16)	−0.05210 (13)	0.07062 (5)	0.0167 (3)	
O5	0.41531 (17)	0.00670 (13)	0.07223 (5)	0.0192 (3)	
H51	0.474 (3)	−0.037 (2)	0.0985 (8)	0.045 (8)*	
H52	0.308 (2)	−0.011 (2)	0.0734 (10)	0.047 (8)*	
O6	0.75988 (16)	0.05770 (13)	0.02674 (5)	0.0134 (3)	
H61	0.790 (3)	0.043 (2)	0.0592 (6)	0.037 (7)*	
H62	0.742 (3)	0.1359 (16)	0.0247 (10)	0.057 (9)*	
O7	1.11543 (18)	0.17328 (13)	0.01223 (5)	0.0188 (3)	
H71	1.062 (3)	0.2483 (18)	0.0034 (9)	0.050 (8)*	
H72	1.221 (2)	0.182 (3)	0.0070 (10)	0.055 (9)*	
O8	0.5861 (2)	−0.10881 (17)	0.14777 (6)	0.0313 (4)	
H81	0.568 (4)	−0.1863 (17)	0.1430 (13)	0.094 (14)*	
H82	0.697 (3)	−0.096 (3)	0.1402 (14)	0.098 (13)*	
O9	0.03985 (19)	−0.38092 (14)	0.01543 (7)	0.0276 (4)	
H91	0.148 (2)	−0.360 (3)	0.0120 (11)	0.070 (10)*	
H92	0.035 (6)	−0.390 (5)	0.0472 (8)	0.20 (3)*	
N1	0.7834 (2)	−0.64050 (15)	0.13131 (6)	0.0173 (4)	
N2	1.2766 (2)	−0.45986 (16)	0.22433 (6)	0.0190 (4)	
C1	0.4744 (2)	−0.26580 (17)	0.01995 (7)	0.0147 (4)	
C2	0.5485 (2)	−0.36230 (17)	0.05081 (7)	0.0134 (4)	
C3	0.4689 (2)	−0.47050 (18)	0.05580 (7)	0.0150 (4)	
H3	0.3633	−0.4813	0.0406	0.018*	
C4	0.5434 (2)	−0.56160 (18)	0.08273 (7)	0.0151 (4)	
H4	0.4869	−0.6324	0.0856	0.018*	
C5	0.7046 (2)	−0.54916 (18)	0.10611 (7)	0.0148 (4)	
C6	0.7801 (2)	−0.43779 (18)	0.10262 (7)	0.0181 (4)	
H6	0.8834	−0.4245	0.1189	0.022*	
C7	0.7036 (2)	−0.34842 (18)	0.07551 (7)	0.0161 (4)	
H7	0.7574	−0.2762	0.0736	0.019*	
C8	0.7200 (3)	−0.76135 (18)	0.12893 (7)	0.0187 (4)	
H8A	0.8115	−0.8161	0.1285	0.022*	
H8B	0.6497	−0.7715	0.0991	0.022*	
C9	0.6227 (3)	−0.7917 (2)	0.17097 (8)	0.0276 (5)	
H9A	0.5955	−0.8745	0.1699	0.041*	
H9B	0.5234	−0.7457	0.1689	0.041*	
H9C	0.6878	−0.7743	0.2007	0.041*	
C10	0.9497 (3)	−0.62677 (19)	0.15495 (8)	0.0212 (5)	
H10A	0.9669	−0.6842	0.1809	0.025*	
H10B	0.9606	−0.5484	0.1691	0.025*	
C11	1.0821 (3)	−0.6433 (2)	0.12076 (9)	0.0298 (5)	
H11A	1.1888	−0.6344	0.1382	0.045*	
H11B	1.0684	−0.5849	0.0957	0.045*	
H11C	1.0727	−0.7210	0.1068	0.045*	
C12	1.0033 (2)	−0.08717 (17)	0.10404 (7)	0.0135 (4)	
C13	1.0729 (2)	−0.18313 (17)	0.13585 (6)	0.0126 (4)	
C14	1.0042 (2)	−0.21580 (17)	0.17791 (6)	0.0131 (4)	
H14	0.9107	−0.1766	0.1861	0.016*	
C15	1.0717 (2)	−0.30486 (18)	0.20755 (7)	0.0152 (4)	
H15	1.0237	−0.3236	0.2355	0.018*	
C16	1.2121 (2)	−0.36804 (17)	0.19621 (7)	0.0152 (4)	
C17	1.2802 (2)	−0.33497 (18)	0.15330 (7)	0.0163 (4)	
H17	1.3729	−0.3743	0.1445	0.020*	
C18	1.2114 (2)	−0.24579 (17)	0.12450 (7)	0.0147 (4)	
H18	1.2586	−0.2265	0.0965	0.018*	
C19	1.2123 (3)	−0.48833 (19)	0.27032 (7)	0.0230 (5)	
H19A	1.2418	−0.5693	0.2790	0.028*	
H19B	1.0931	−0.4835	0.2662	0.028*	
C20	1.2764 (3)	−0.4072 (2)	0.31101 (8)	0.0293 (5)	
H20A	1.2227	−0.4254	0.3393	0.044*	
H20B	1.2542	−0.3264	0.3018	0.044*	
H20C	1.3931	−0.4182	0.3178	0.044*	
C21	1.4284 (3)	−0.51786 (19)	0.21392 (8)	0.0223 (5)	
H21A	1.4247	−0.5299	0.1794	0.027*	
H21B	1.4330	−0.5954	0.2290	0.027*	
C22	1.5858 (3)	−0.4513 (2)	0.23068 (9)	0.0343 (6)	
H22A	1.6787	−0.4933	0.2206	0.051*	
H22B	1.5964	−0.4453	0.2652	0.051*	
H22C	1.5815	−0.3733	0.2169	0.051*	

### Atomic displacement parameters (Å^2^)

**Table:**

	*U*^11^	*U*^22^	*U*^33^	*U*^12^	*U*^13^	*U*^23^
Mn1	0.0105 (2)	0.0107 (2)	0.01513 (19)	0.00024 (17)	0.00158 (15)	0.00282 (17)
Mn2	0.0106 (2)	0.0136 (2)	0.01241 (19)	0.00140 (17)	0.00160 (15)	0.00314 (17)
O1	0.0156 (7)	0.0125 (8)	0.0219 (7)	0.0011 (6)	0.0006 (6)	0.0018 (6)
O2	0.0145 (7)	0.0122 (8)	0.0294 (8)	0.0013 (6)	0.0073 (6)	0.0067 (6)
O3	0.0157 (7)	0.0159 (8)	0.0172 (7)	0.0028 (6)	0.0028 (6)	0.0034 (6)
O4	0.0148 (7)	0.0202 (8)	0.0151 (7)	−0.0004 (6)	0.0017 (5)	0.0059 (6)
O5	0.0142 (7)	0.0260 (9)	0.0175 (7)	0.0000 (7)	0.0023 (6)	0.0031 (6)
O6	0.0131 (7)	0.0132 (8)	0.0139 (7)	0.0011 (6)	0.0008 (5)	0.0016 (6)
O7	0.0146 (8)	0.0148 (8)	0.0278 (8)	0.0007 (6)	0.0053 (6)	0.0022 (6)
O8	0.0217 (9)	0.0393 (12)	0.0325 (9)	−0.0029 (8)	0.0008 (7)	0.0125 (8)
O9	0.0172 (8)	0.0201 (9)	0.0458 (10)	0.0001 (7)	0.0056 (7)	0.0033 (8)
N1	0.0199 (9)	0.0104 (9)	0.0210 (9)	0.0007 (7)	−0.0011 (7)	0.0030 (7)
N2	0.0240 (9)	0.0180 (9)	0.0155 (8)	0.0083 (8)	0.0043 (7)	0.0051 (7)
C1	0.0194 (10)	0.0109 (11)	0.0146 (9)	0.0014 (8)	0.0069 (8)	−0.0004 (8)
C2	0.0152 (10)	0.0114 (10)	0.0144 (9)	0.0035 (8)	0.0048 (8)	0.0006 (8)
C3	0.0147 (10)	0.0155 (11)	0.0152 (9)	0.0021 (8)	0.0036 (8)	0.0006 (8)
C4	0.0177 (10)	0.0118 (11)	0.0164 (9)	−0.0006 (8)	0.0044 (8)	0.0006 (8)
C5	0.0195 (10)	0.0130 (10)	0.0126 (9)	0.0026 (8)	0.0043 (8)	0.0005 (8)
C6	0.0152 (10)	0.0171 (12)	0.0212 (10)	−0.0004 (9)	−0.0016 (8)	0.0014 (8)
C7	0.0185 (10)	0.0106 (10)	0.0194 (10)	−0.0013 (8)	0.0034 (8)	0.0008 (8)
C8	0.0238 (11)	0.0104 (11)	0.0218 (10)	0.0042 (9)	0.0017 (9)	0.0014 (8)
C9	0.0349 (13)	0.0199 (13)	0.0294 (12)	−0.0011 (10)	0.0104 (10)	0.0038 (10)
C10	0.0221 (11)	0.0165 (12)	0.0234 (10)	0.0015 (9)	−0.0050 (9)	0.0044 (9)
C11	0.0230 (12)	0.0307 (14)	0.0354 (13)	0.0032 (10)	0.0021 (10)	0.0035 (11)
C12	0.0147 (10)	0.0114 (10)	0.0138 (9)	−0.0025 (8)	−0.0012 (7)	−0.0011 (7)
C13	0.0146 (10)	0.0105 (10)	0.0120 (9)	−0.0013 (8)	−0.0020 (7)	0.0001 (7)
C14	0.0147 (10)	0.0106 (10)	0.0141 (9)	−0.0005 (8)	0.0008 (7)	−0.0011 (7)
C15	0.0199 (10)	0.0152 (11)	0.0108 (9)	−0.0016 (8)	0.0028 (8)	0.0002 (8)
C16	0.0202 (11)	0.0121 (11)	0.0130 (9)	0.0021 (8)	−0.0007 (8)	0.0012 (8)
C17	0.0170 (10)	0.0172 (11)	0.0150 (9)	0.0036 (8)	0.0029 (8)	−0.0008 (8)
C18	0.0168 (10)	0.0168 (11)	0.0108 (9)	−0.0003 (8)	0.0025 (7)	0.0019 (7)
C19	0.0281 (12)	0.0221 (13)	0.0193 (10)	0.0117 (10)	0.0040 (9)	0.0082 (9)
C20	0.0313 (13)	0.0348 (15)	0.0218 (11)	0.0047 (11)	0.0018 (10)	0.0026 (10)
C21	0.0275 (12)	0.0201 (13)	0.0196 (10)	0.0105 (9)	0.0039 (9)	0.0043 (9)
C22	0.0265 (13)	0.0452 (16)	0.0318 (13)	0.0062 (12)	0.0059 (10)	0.0009 (12)

### Geometric parameters (Å, °)

**Table:**

Mn1—O2	2.1071 (14)	C6—C5	1.408 (3)
Mn1—O2^i^	2.1071 (14)	C6—C7	1.373 (3)
Mn1—O5	2.1932 (14)	C6—H6	0.9300
Mn1—O5^i^	2.1932 (14)	C7—H7	0.9300
Mn1—O6	2.2725 (13)	C8—C9	1.518 (3)
Mn1—O6^i^	2.2725 (13)	C8—H8A	0.9700
Mn2—O4	2.1120 (13)	C8—H8B	0.9700
Mn2—O4^ii^	2.1120 (13)	C9—H9A	0.9600
Mn2—O6	2.2594 (13)	C9—H9B	0.9600
Mn2—O6^ii^	2.2594 (13)	C9—H9C	0.9600
Mn2—O7	2.1835 (14)	C10—C11	1.520 (3)
Mn2—O7^ii^	2.1835 (14)	C10—H10A	0.9700
O1—C1	1.263 (2)	C10—H10B	0.9700
O2—C1	1.279 (2)	C11—H11A	0.9600
O3—C12	1.263 (2)	C11—H11B	0.9600
O4—C12	1.278 (2)	C11—H11C	0.9600
O5—H51	0.970 (16)	C12—C13	1.478 (3)
O5—H52	0.903 (17)	C13—C18	1.396 (3)
O6—H61	0.931 (16)	C14—C13	1.398 (3)
O6—H62	0.896 (18)	C14—C15	1.381 (3)
O7—H71	0.973 (16)	C14—H14	0.9300
O7—H72	0.894 (17)	C15—H15	0.9300
O8—H81	0.895 (17)	C16—C15	1.412 (3)
O8—H82	0.957 (19)	C16—C17	1.417 (3)
O9—H91	0.930 (17)	C17—C18	1.373 (3)
O9—H92	0.90 (2)	C17—H17	0.9300
N1—C5	1.372 (2)	C18—H18	0.9300
N1—C8	1.458 (3)	C19—C20	1.511 (3)
N1—C10	1.457 (3)	C19—H19A	0.9700
N2—C16	1.373 (2)	C19—H19B	0.9700
N2—C19	1.468 (2)	C20—H20A	0.9600
N2—C21	1.455 (3)	C20—H20B	0.9600
C1—C2	1.480 (3)	C20—H20C	0.9600
C2—C3	1.397 (3)	C21—C22	1.521 (3)
C2—C7	1.389 (3)	C21—H21A	0.9700
C3—H3	0.9300	C21—H21B	0.9700
C4—C3	1.379 (3)	C22—H22A	0.9600
C4—C5	1.416 (3)	C22—H22B	0.9600
C4—H4	0.9300	C22—H22C	0.9600
			
O2^i^—Mn1—O2	180.00 (11)	C6—C7—C2	122.17 (19)
O2^i^—Mn1—O5	90.28 (5)	C6—C7—H7	118.9
O2—Mn1—O5	89.72 (5)	N1—C8—C9	112.74 (17)
O2^i^—Mn1—O5^i^	89.72 (5)	N1—C8—H8A	109.0
O2—Mn1—O5^i^	90.28 (5)	N1—C8—H8B	109.0
O2^i^—Mn1—O6	89.78 (5)	C9—C8—H8A	109.0
O2—Mn1—O6	90.22 (5)	C9—C8—H8B	109.0
O2—Mn1—O6^i^	89.78 (5)	H8A—C8—H8B	107.8
O2^i^—Mn1—O6^i^	90.22 (5)	C8—C9—H9A	109.5
O5—Mn1—O5^i^	180.00 (7)	C8—C9—H9B	109.5
O5—Mn1—O6	93.27 (5)	C8—C9—H9C	109.5
O5^i^—Mn1—O6	86.73 (5)	H9A—C9—H9B	109.5
O5—Mn1—O6^i^	86.73 (5)	H9A—C9—H9C	109.5
O5^i^—Mn1—O6^i^	93.27 (5)	H9B—C9—H9C	109.5
O6—Mn1—O6^i^	180.00 (10)	N1—C10—C11	113.00 (18)
O4—Mn2—O4^ii^	180.00 (8)	N1—C10—H10A	109.0
O4—Mn2—O6^ii^	90.00 (5)	N1—C10—H10B	109.0
O4^ii^—Mn2—O6^ii^	90.00 (5)	C11—C10—H10A	109.0
O4—Mn2—O6	90.00 (5)	C11—C10—H10B	109.0
O4^ii^—Mn2—O6	90.00 (5)	H10A—C10—H10B	107.8
O4—Mn2—O7	90.10 (5)	C10—C11—H11A	109.5
O4^ii^—Mn2—O7	89.90 (5)	C10—C11—H11B	109.5
O4—Mn2—O7^ii^	89.90 (5)	C10—C11—H11C	109.5
O4^ii^—Mn2—O7^ii^	90.10 (5)	H11A—C11—H11B	109.5
O6^ii^—Mn2—O6	180.00 (6)	H11A—C11—H11C	109.5
O7—Mn2—O7^ii^	180.00 (8)	H11B—C11—H11C	109.5
O7—Mn2—O6^ii^	86.29 (5)	O3—C12—O4	122.35 (18)
O7^ii^—Mn2—O6^ii^	93.71 (5)	O3—C12—C13	120.23 (17)
O7—Mn2—O6	93.71 (5)	O4—C12—C13	117.42 (17)
O7^ii^—Mn2—O6	86.29 (5)	C14—C13—C12	122.31 (17)
C1—O2—Mn1	127.87 (12)	C18—C13—C12	120.51 (17)
C12—O4—Mn2	127.31 (12)	C18—C13—C14	117.18 (17)
Mn1—O5—H51	120.1 (16)	C13—C14—H14	119.2
Mn1—O5—H52	114.8 (18)	C15—C14—C13	121.64 (18)
H52—O5—H51	105 (2)	C15—C14—H14	119.2
Mn1—O6—H61	114.7 (15)	C14—C15—C16	121.24 (18)
Mn1—O6—H62	97.0 (18)	C14—C15—H15	119.4
Mn2—O6—Mn1	128.35 (6)	C16—C15—H15	119.4
Mn2—O6—H61	96.4 (15)	N2—C16—C15	121.84 (17)
Mn2—O6—H62	113.8 (19)	N2—C16—C17	121.39 (18)
H61—O6—H62	106 (2)	C15—C16—C17	116.73 (18)
Mn2—O7—H71	124.4 (16)	C18—C17—C16	121.00 (18)
Mn2—O7—H72	118.9 (19)	C18—C17—H17	119.5
H72—O7—H71	106 (2)	C16—C17—H17	119.5
H81—O8—H82	105 (3)	C17—C18—C13	122.20 (18)
H91—O9—H92	105 (3)	C17—C18—H18	118.9
C5—N1—C8	122.25 (17)	C13—C18—H18	118.9
C5—N1—C10	121.36 (17)	N2—C19—C20	113.32 (18)
C10—N1—C8	115.50 (16)	N2—C19—H19A	108.9
C16—N2—C19	120.96 (16)	N2—C19—H19B	108.9
C16—N2—C21	120.77 (16)	C20—C19—H19A	108.9
C21—N2—C19	117.38 (16)	C20—C19—H19B	108.9
O1—C1—O2	121.96 (18)	H19A—C19—H19B	107.7
O1—C1—C2	120.75 (17)	C19—C20—H20A	109.5
O2—C1—C2	117.28 (17)	C19—C20—H20B	109.5
C3—C2—C1	122.32 (17)	C19—C20—H20C	109.5
C7—C2—C1	120.34 (18)	H20A—C20—H20B	109.5
C7—C2—C3	117.33 (18)	H20A—C20—H20C	109.5
C2—C3—H3	119.3	H20B—C20—H20C	109.5
C4—C3—C2	121.48 (18)	N2—C21—C22	115.19 (19)
C4—C3—H3	119.3	N2—C21—H21A	108.5
C3—C4—C5	121.09 (19)	N2—C21—H21B	108.5
C3—C4—H4	119.5	C22—C21—H21A	108.5
C5—C4—H4	119.5	C22—C21—H21B	108.5
N1—C5—C4	121.91 (18)	H21A—C21—H21B	107.5
N1—C5—C6	121.37 (18)	C21—C22—H22A	109.5
C6—C5—C4	116.72 (18)	C21—C22—H22B	109.5
C5—C6—H6	119.5	C21—C22—H22C	109.5
C7—C6—C5	121.07 (18)	H22A—C22—H22B	109.5
C7—C6—H6	119.5	H22A—C22—H22C	109.5
C2—C7—H7	118.9	H22B—C22—H22C	109.5
			
O5—Mn1—O2—C1	60.63 (16)	C16—N2—C19—C20	−79.2 (2)
O5^i^—Mn1—O2—C1	−119.37 (16)	C21—N2—C19—C20	90.1 (2)
O6—Mn1—O2—C1	153.90 (16)	C16—N2—C21—C22	78.8 (2)
O6^i^—Mn1—O2—C1	−26.10 (16)	C19—N2—C21—C22	−90.5 (2)
O2^i^—Mn1—O6—Mn2	−125.04 (8)	O1—C1—C2—C3	12.0 (3)
O2—Mn1—O6—Mn2	54.96 (8)	O1—C1—C2—C7	−169.14 (18)
O5—Mn1—O6—Mn2	144.69 (8)	O2—C1—C2—C3	−168.63 (17)
O5^i^—Mn1—O6—Mn2	−35.31 (8)	O2—C1—C2—C7	10.2 (3)
O6^ii^—Mn2—O4—C12	−151.88 (16)	C1—C2—C3—C4	176.60 (18)
O6—Mn2—O4—C12	28.12 (16)	C7—C2—C3—C4	−2.3 (3)
O7—Mn2—O4—C12	121.83 (16)	C1—C2—C7—C6	−176.75 (18)
O7^ii^—Mn2—O4—C12	−58.17 (16)	C3—C2—C7—C6	2.2 (3)
O4—Mn2—O6—Mn1	−124.77 (8)	C5—C4—C3—C2	−0.5 (3)
O4^ii^—Mn2—O6—Mn1	55.23 (8)	C3—C4—C5—N1	−177.13 (18)
O7—Mn2—O6—Mn1	145.13 (8)	C3—C4—C5—C6	3.4 (3)
O7^ii^—Mn2—O6—Mn1	−34.87 (8)	C7—C6—C5—N1	176.98 (18)
Mn1—O2—C1—O1	31.0 (3)	C7—C6—C5—C4	−3.5 (3)
Mn1—O2—C1—C2	−148.33 (13)	C5—C6—C7—C2	0.8 (3)
Mn2—O4—C12—O3	−36.7 (3)	O3—C12—C13—C14	−11.0 (3)
Mn2—O4—C12—C13	142.49 (14)	O3—C12—C13—C18	168.54 (18)
C8—N1—C5—C4	10.9 (3)	O4—C12—C13—C14	169.77 (17)
C8—N1—C5—C6	−169.68 (18)	O4—C12—C13—C18	−10.7 (3)
C10—N1—C5—C4	179.60 (18)	C12—C13—C18—C17	179.60 (18)
C10—N1—C5—C6	−0.9 (3)	C14—C13—C18—C17	−0.8 (3)
C5—N1—C8—C9	−97.7 (2)	C15—C14—C13—C12	−179.32 (18)
C10—N1—C8—C9	92.9 (2)	C15—C14—C13—C18	1.1 (3)
C5—N1—C10—C11	−82.3 (2)	C13—C14—C15—C16	−0.9 (3)
C8—N1—C10—C11	87.2 (2)	N2—C16—C15—C14	−177.38 (18)
C19—N2—C16—C17	175.89 (19)	C17—C16—C15—C14	0.4 (3)
C19—N2—C16—C15	−6.5 (3)	N2—C16—C17—C18	177.68 (19)
C21—N2—C16—C15	−175.42 (19)	C15—C16—C17—C18	−0.1 (3)
C21—N2—C16—C17	6.9 (3)	C16—C17—C18—C13	0.3 (3)

Symmetry codes: (i) −*x*+1, −*y*, −*z*; (ii) −*x*+2, −*y*, −*z*.

### Hydrogen-bond geometry (Å, °)

**Table:**

*D*—H···*A*	*D*—H	H···*A*	*D*···*A*	*D*—H···*A*
O5—H51···O8	0.97 (2)	1.77 (2)	2.738 (2)	177 (2)
O5—H52···O4^iii^	0.90 (2)	1.85 (2)	2.7457 (19)	175 (3)
O6—H61···O3	0.93 (2)	1.78 (2)	2.651 (2)	156 (2)
O6—H62···O1^i^	0.90 (2)	1.76 (2)	2.608 (2)	156 (3)
O7—H71···O9^i^	0.97 (2)	1.77 (2)	2.739 (2)	177 (1)
O7—H72···O2^ii^	0.89 (2)	1.85 (2)	2.733 (2)	170 (3)
O8—H82···O3	0.96 (3)	1.77 (3)	2.694 (2)	160 (3)
O9—H91···O1	0.93 (2)	1.80 (2)	2.692 (2)	160 (3)
C6—H6···Cg2	0.93	2.91	3.764 (2)	154
C19—H19A···Cg2^iv^	0.97	2.90	3.830 (2)	162

Symmetry codes: (iii) *x*−1, *y*, *z*; (i) −*x*+1, −*y*, −*z*; (ii) −*x*+2, −*y*, −*z*;  (iv) −*x*+1/2, *y*−1/2, −*z*+1/2.
